# Are the knee and ankle angles at contact related to the tendon properties of lower limbs in long distance runners?

**DOI:** 10.1186/s40064-016-1797-1

**Published:** 2016-02-24

**Authors:** Keitaro Kubo, Daisuke Miyazaki, Kenji Yamada, Shozo Shimoju, Naoya Tsunoda

**Affiliations:** Department of Life Science (Sports Sciences), The University of Tokyo, Komaba 3-8-1, Meguro-ku, Tokyo, 153-8902 Japan; Faculty of Physical Education, Kokushikan University, Tokyo, Japan

**Keywords:** Tendon stiffness, Knee extensors, Plantar flexors, Ultrasonography

## Abstract

The purpose of this study was to investigate whether the knee and ankle angles at contact during running were related to the elastic properties of tendon structures in knee extensors and plantar flexors and performance in trained long distance runners. Thirty-two highly trained male long distance runners participated in this study. Elongation of tendon structures in knee extensors and plantar flexors were measured using ultrasonography while subjects performed ramp isometric contractions up to the voluntary maximum. The relationship between estimated muscle force and tendon elongation was fit to a linear regression, the slope of which was defined as the stiffness of tendon structures. Knee and ankle angles at contact during running were determined at a speed of 18 km/h on a treadmill. Knee and ankle angles at contact were not correlated to the stiffness of tendon structures in knee extensors and plantar flexors. In addition, the best official record in a 5000-m race was not significantly correlated to knee and ankle joint angles at contact. In conclusion, knee and ankle angles at contact were not related to the elastic properties of tendon structures in knee extensors and plantar flexor and the performance of long distance running.

## Background

Recently, the foot strike pattern during running is receiving a lot of attentions among recreational and competitive runners (e.g., Lieberman et al. 2010). Previous studies showed that a higher percentage of forefoot strike runners, in which the ball of the foot contacts ground before the heel comes down, was observed among elite long distance runners (Hasegawa et al. 2007; Kasmer et al. 2013; Larson et al. 2011). On the contrary, rearfoot strike runners, in which the heel first contacts the ground, were more economical than forefoot strike runners according to the other studies (Ogueta-Alday et al. 2014; Williams and Cavanagh 1987). To date, therefore, perspectives on the effects of foot strike pattern on the running performance and economy have not yet been unified. On the other hand, several reports have used ultrasonography to investigate the relationship between the tendon properties of lower limbs and the running performance and economy (Arampatzis et al. 2006; Fletcher et al. 2010; Kubo et al. 2010, 2015a). For example, Fletcher et al. (2010) demonstrated that higher stiffness of the Achilles tendon was associated with lower oxygen consumption. Considering these findings, the foot strike pattern may be related to the elastic properties of the Achilles tendon. However, our recent study demonstrated that no differences in the morphological or elastic properties of the Achilles tendon and the best official record in a 5000-m race were found among the foot strike patterns of long distance runners (Kubo et al. 2015b). Accordingly, we expected that the ankle angle at contact (similar to the foot strike pattern) was not related to the Achilles tendon properties and running performance.

The knee joint angle at ground contact during running has so far been less noticed compared to the foot strike pattern as mentioned above. Some previous studies showed that knee joint angle at contact during running influenced the peak vertical ground reaction impact force (Derrick 2004; Potthast et al. 2010). According to these findings (Derrick 2004; Potthast et al. 2010), increasing knee flexion angle at contact reduced the peak vertical ground reaction impact force. Therefore, it is likely that imposed mechanical stress on lower limbs was greater in runners with the knee straight at contact than in ones with the knee flexed. From the viewpoint of biomechanics, the runners with the knee straight at contact would have an advantage in running performance and economy, because an up-and-down motion of center of mass would be smaller during running. More recently, we found that better long distance runners had stiffer tendon structures in their knee extensors due to the greater mechanical stresses imposed on the knee extensors during long-term running training (Kubo et al. 2015b). Considering these points, the runners contacting more extended knee position would have stiffer tendon structures in knee extensors and exhibit the higher running performance.

The running performance and economy were partially affected by the running form and gait patterns (Anderson 1996; Morgan et al. 1989). If the running form (e.g., joint angles at contact) is related to the tendon properties and running performance, these observations would be useful to instruct in running form (taking the tendon properties of each runner into consideration) in track and field. In the present study, we investigated whether the knee and ankle angles at contact during running were related to the elastic properties of tendon structures in knee extensors and plantar flexors and performance in trained long distance runners. We hypothesized that the elastic properties of tendon structures and the performance were related to the knee joint angle at contact, but not to the ankle joint angle.

## Methods

### Subjects

Thirty-two highly trained male long distance runners (age: 20.2 ± 1.0 years, height: 169.9 ± 5.2 cm, body mass: 57.2 ± 4.8 kg, mean ± SD) participated in this study. They had participated in competitive meets at the regional or intercollegiate level within the preceding year. The best official record in a 5000-m race within 1 year prior to these tests ranged from 14:11 to 16:15 (14:54 ± 0:26) (min:s). The subjects were fully informed of the procedures to be utilized as well as the purpose of this study. Written informed consent was obtained from all subjects. This study was approved by the office of the Department of Sports Sciences, The University of Tokyo, and complied with their requirements for human experimentation.

### Knee and ankle angles at contact during running

The knee and ankle joint angles at contact during running were determined at sub-maximal velocity (18 km/h) on a treadmill (AR-100, Minato Medical Science, Osaka, Japan). After a warm-up period of 4 min at a running velocity of 10 km/h, the subjects ran at three sub-maximal velocities (14, 16, and 18 km/h) for 4 min. At the final phase (approximately 30 s) at a speed of 18 km/h, a sagittal image of the entire stance phase of the leg of runners was obtained using a high-speed video camera (sampling rate 250 Hz; VCC-H1600C, Digimo, Tokyo, Japan) placed on the right side of the treadmill. Reflective markers were placed on trochanter major, the center of rotation of the knee, lateral malleolus tip, and fifth metararsal head. These points were digitized and filtered with Butterworth fourth-order filter (cut-off frequency 10 Hz) using digitizing software (Frame-DIAS VI, DKH Inc., Japan) to calculate the knee (full extension 0°) and ankle (anatomical position 0° with positive values of plantar flexion) angles at contact. The data (knee and ankle joint angles) of five steps have been analyzed and averaged for each subject.

### Elastic properties of tendon structures

Maximal voluntary isometric contraction (MVC) was measured by means of specially designed dynamometers (Applied Office, Tokyo, Japan) for knee extension and plantar flexion. During each task, subjects exerted isometric torque from zero (relax) to MVC within 5 s. Details of the posture of the subjects and setup have been described elsewhere (Kubo et al. 2010). Elongations of tendon structures for knee extensors and plantar flexors were assessed during isometric contractions. An ultrasonic apparatus (SSD-6500, Aloka, Tokyo, Japan) with an electronic linear array probe was used to obtain longitudinal ultrasonic images of vastus lateralis and medial gastrocnemius muscles by procedures described previously (Kubo et al. 2010). Ultrasonic images were recorded on videotape at 30 Hz and synchronized with recordings of a clock timer for subsequent analysis. The point at which one fascicle was attached to the aponeurosis was visualized on ultrasonic images. The displacement of this point is considered to indicate lengthening of the deep aponeurosis and distal tendon. To correct measurements taken for tendon and aponeurosis elongation, additional measurements were taken under passive conditions (Kubo et al. 2010). For each subject, the displacement of each site obtained from ultrasonic images could be corrected for that attributed to joint rotation alone. In this study, only values corrected for angular rotation were reported.

Torque (TQ) measured during isometric contractions was converted to muscle force (Fm) by the following equation (Kubo et al. 2010):

$${\text{Fm}} = {\text{k}} \cdot {\text{TQ}} \cdot {\text{MA}}^{ - 1}$$

where k is the relative contribution of physiological cross-sectional area in each vastus lateralis muscle within knee extensors and medial gastrocnemius muscle within plantar flexors, and MA is the moment arm length in each quadriceps femoris muscles at 90° and triceps surae muscle at 90°, which was estimated from the limb length of each subject (Kubo et al. 2010). In this study, Fm and tendon elongation above 50 % of MVC were fitted to a linear regression equation, the slope of which was adopted as stiffness (Kubo et al. 2010).

### Statistics

Descriptive data represent mean ± SD. Peason product-moment correlation coefficients were computed to assess the relationships between the measured variables. The level of significance was set at p < 0.05.

## Results

**Fig. 1:**
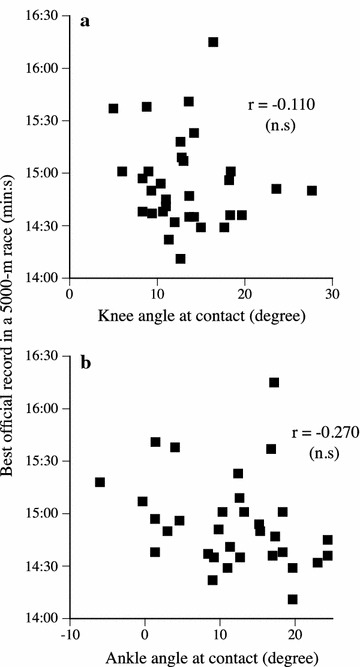
Relationships between the best official record in a 5000-m race and the knee (**a**) and ankle (**b**) joint angles at contact

On average, the knee and ankle joint angles at contact were 13.3° ± 4.9° (range 5.0°–27.7°) and 11.7° ± 7.6° (range −6.0° to 24.3°), respectively. The knee joint angle at contact was not correlated to maximal elongation and stiffness of the tendon structures in knee extensors (Table 1). Similarly, no significant correlations were found between the ankle joint angle at contact and the measured variables (maximal elongation and stiffness) of the tendon structures in plantar flexors (Table 1). Furthermore, the running performance (best official record in a 5000-m race) was not significantly correlated to both the knee and ankle joint angles at contact (Fig. 1).

**Table 1:** Correlation coefficient between the knee and ankle joint angles at contact and measured variables

	Versus maximal elongation	Versus tendon stiffness
Knee angle	−0.061	0.062
Ankle angle	−0.250	−0.052

## Discussion

Previous studies showed that the knee angle at contact was related to the peak vertical ground reaction impact force (Derrick 2004; Potthast et al. 2010). On the other hand, it has been reported that elite long distance runners generally run 90–150 km a week (Holmich et al. 1988). It is possible that the joint compressive forces, but not knee extensor moment, are increased when the vertical ground reaction force is greater at contact during running. According to the previous findings (Kulmala et al. 2013; Williams et al. 2000), however, the rearfoot strike runners landed with more extended knee position (although the present result was different) and exhibited greater knee extensor moment. Hence, we may say that imposed mechanical stress on lower limbs was greater in runners with the knee straight at contact than in ones with the knee flexed. Previous researchers demonstrated that mechanical stress was found to be important for changes in tendon stiffness (Arampatzis et al. 2007; Kubo et al. 2006). Therefore, it is possible to substantiate the hypothesis that the tendon structures in knee extensors would be stiffer for runners contacting more extended knee position. In the present study, however, this hypothesis was rejected. Our previous studies showed that the tendon stiffness increased markedly after isometric training of a longer duration (Kubo et al. 2001, 2009), although the tendon stiffness did not change after ballistic and plyometric training regimens (Kubo et al. 2001, 2007). Taking these findings into account together with the present result, the mechanical stresses cyclically imposed on the knee extensors at contact with extended knee position during endurance running might be not enough to stiffen the tendon structures in knee extensors.

Our recent study showed that no differences in the morphological or elastic properties of the Achilles tendon among the foot strike patterns of long distance runners (Kubo et al. 2015a). Furthermore, we confirmed that ankle joint angle at contact was not related to the maximal elongation and stiffness of tendon structures in plantar flexors. To date, previous findings concerning the relationship between foot strike patters and running performance and economy are conflicting (Hasegawa et al. 2007; Kasmer et al. 2013; Larson et al. 2011; Ogueta-Alday et al. 2014; Williams and Cavanagh 1987). On the other hand, the findings of musculoskeletal modeling studies showed that the lower metabolic energy expenditure by muscle fibers during walking and running was associated with the elastic energy stored in the Achilles tendon (Farris and Sawicki 2012; Hof et al. 2002; Sasaki and Neptune 2006). Furthermore, we reported previously that better long distance runners had more compliant tendon structures in plantar flexors (Kubo et al. 2010, 2015a). Therefore, we may say that the elastic properties of Achilles tendon contributed to store the elastic energy during endurance running irrespective of ankle joint angle at contact (the foot strike patterns).

Runners with the knee straight at contact would have an advantage in running performance and economy, since an up-and down motion of center of mass was smaller during running. Indeed, Leskinen et al. (2009) reported that during competitions the minimum knee angle in the stance phase was smaller, i.e., more knee extended position, in five elite 1500-m runners (seasonal best: 3 min 35.6 s ± 2.6 s) than in six national-standard ones (seasonal best: 3 min 49.2 s ± 3.2 s). Although previous findings concerning the relationship between the foot strike patterns and running performance are conflicting, some researchers demonstrated that forefoot strike runners was faster than rearfoot strike runners (Hasegawa et al. 2007; Kasmer et al. 2013; Larson et al. 2011). In the present study, however, the best official record in a 5000-m race was not significantly correlated to both the knee and ankle joint angles at contact (Fig. 1). Therefore, it is likely that the knee and ankle joint angles at contact did not affect the performance of long distance runners.

**Fig. 2:**
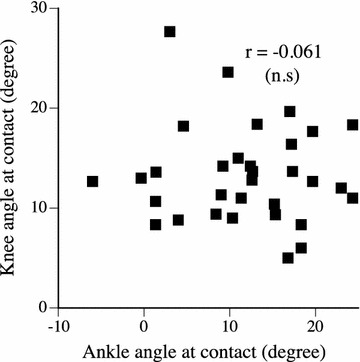
The relationships between the knee and ankle joint angles at contact

Some previous studies demonstrated that the forefoot strike runners land with a more flexed knee joint compared to the rearfoot strike runners (Lieberman et al. 2010; Shin et al. 2013; Williams et al. 2000). Williams et al. (2000) stated that increasing the knee flexion angle at ground contact compensated the plantar flexion ankle in forefoot strike running, and thus adjustments in the knee and ankle motions helped to minimize the vertical movement of the center of mass to minimize energy consumption during running. In the present study, however, there was no significant correlation between the knee and ankle joint angles at ground contact (Fig. 2). The present result disagreed with the previous finding (Lieberman et al. 2010; Shin et al. 2013; Williams et al. 2000). Possible reason for the discrepancy is the differences in the competition ability in the long distance race event between the present study (highly trained runners) and other previous studies (including recreational runners).

## Conclusion

The results of this study indicated that the elastic properties of tendon structures in knee extensors and plantar flexors did not affect the knee and ankle joint angles at contact during endurance running. Furthermore, the knee and ankle angles at contact were not related to the performance of long distance runners. Therefore, it may be that the knee and ankle joint angles at contact are related to the other parameters, e.g., mechanical properties of muscle, flexibility of joint.

## References

[CR1] Anderson T (1996). Biomechanics and running economy. Sports Med.

[CR2] Arampatzis A, DeMonte G, Karamanidis K, Morey-Klapsing G, Stafilidis S, Bruggemann GP (2006). Influence of the muscle-tendon unit’s mechanical and morphological properties on running economy. J Exp Biol.

[CR3] Arampatzis A, Karamanidis K, Albracht K (2007). Adaptational responses of the human Achilles tendon by modulation of the applied cyclic strain magnitude. J Exp Biol.

[CR4] Derrick TR (2004). The effects of knee contact angle on impact forces and accelerations. Med Sci Sports Exerc.

[CR5] Farris DJ, Sawicki GS (2012). Human medial gastrocnemius force–velocity behavior shifts with locomotion speed and gait. Proc Natl Acad Sci.

[CR6] Fletcher JR, Esau SP, MacIntosh BR (2010). Changes in tendon stiffness and running economy in highly trained distance runners. Eur J Appl Physiol.

[CR7] Hasegawa H, Yamauchi T, Kraemer WJ (2007). Foot strike patterns of runners at the 15-km point during an elite-level half marathon. J Strength Cond Res.

[CR8] Hof AL, Van Zandwijk JP, Bobbert MF (2002). Mechanics of human triceps surae muscle in walking, running and jumping. Acta Physiol Scand.

[CR9] Holmich P, Darre E, Jahnsen F, Harting-Jensen T (1988). The elite marathon runner: problems during and after competition. Br J Sports Med.

[CR10] Kasmer ME, Liu XC, Roberts KG, Valadao JM (2013). Foot-strike pattern and performance in a marathon. Int J Sports Physiol Perform.

[CR11] Kubo K, Kanehisa H, Fukunaga T (2001). Effects of different duration isometric contractions on tendon elasticity of human quadriceps muscles. J Phsiol.

[CR12] Kubo K, Komuro T, Ishiguro N, Tunoda N, Sato Y, Ishii N, Kanehisa H, Fukunaga T (2006). Effects of low load resistance training with vascular occlusion on the mechanical properties of muscle and tendon. J Appl Biomech.

[CR13] Kubo K, Morimoto M, Komuro T, Yata H, Tsunoda N, Kanehisa H, Fukunaga T (2007). Effects of plyometric and weight training on muscle-tendon complex and jump performance. Med Sci Sports Exerc.

[CR14] Kubo K, Ikebukuro T, Yaeshima K, Yata H, Tsunoda N, Kanehisa H (2009). Effects of static and dynamic training on the stiffness and blood volume of tendon in vivo. J Appl Physiol.

[CR15] Kubo K, Tabata T, Ikebukuro T, Igarashi K, Yata H, Tsunoda N (2010). Effects of mechanical properties of muscle and tendon on performance in long distance runners. Eur J Appl Physiol.

[CR16] Kubo K, Miyazaki D, Tanaka S, Shimoju S, Tsunoda N (2015). Relationship between Achilles tendon properties and footstrike patterns in long distance runners. J Sports Sci.

[CR17] Kubo K, Miyazaki D, Shimoju S, Tsunoda N (2015). Relationship between elastic properties of tendon structures and performance in long distance runners. Eur J Appl Physiol.

[CR18] Kulmala JP, Avela J, Pasanen K, Parkkari J (2013). Forefoot strikers exhibit lower running-induced knee loading than rearfoot strikers. Med Sci Sports Exerc.

[CR19] Larson P, Higgins E, Kaminski J, Decker T, Preble J, Lyons D, McIntyre K, Normile A (2011). Foot strike patterns of recreational and sub-elite runners in a long-distance road race. J Sports Sci.

[CR20] Leskinen A, Hakkinen K, Virmavirta M, Isolehto J, Kyrolainen H (2009). Comparison of running kinematics between elite and national-standard 1500-m runners. Sports Biomech.

[CR21] Lieberman DE, Venkadesan M, Werbel WA, Daoud AI, D’Andrea S, Davis IS, Mang’eni RO, Pitsiladis Y (2010). Foot strike patterns and collision forces in habitually barefoot versus shod runners. Nature.

[CR22] Morgan DW, Martin PE, Krahenbuhl GS (1989). Factors affecting running economy. Sports Med.

[CR23] Ogueta-Alday A, Rodriguez-Marroyo JA, Garcia-Lopez J (2014). Rearfoot striking runners are more economical than midfoot strikers. Med Sci Sports Exerc.

[CR24] Potthast W, Bruggemann GP, Lundberg A, Arndt A (2010). The influences of impact interface, muscle activity, and knee angle on impact forces and tibial and femoral accelerations occurring after external impacts. J Appl Biomech.

[CR25] Sasaki K, Neptune RR (2006). Muscle mechanical work and elastic energy utilization during walking and running near the preferred gait transition speed. Gait Posture.

[CR26] Shin Y, Lin KL, Shiang TY (2013). Is the foot striking pattern more important than barefoot or shod conditions in running?. Gait Posture.

[CR27] Williams KR, Cavanagh PR (1987). Relationship between distance running mechanics, running economy, and performance. J Appl Physiol.

[CR28] Williams DS, McClay IS, Manal KT (2000). Lower extremity mechanics in runners with a converted forefoot strike pattern. J Appl Biomech.

